# Public Health Response to Clusters of Rapid HIV Transmission Among Hispanic or Latino Gay, Bisexual, and Other Men Who Have Sex with Men — Metropolitan Atlanta, Georgia, 2021–2022

**DOI:** 10.15585/mmwr.mm7210a3

**Published:** 2023-03-10

**Authors:** Carlos Saldana, David C. Philpott, Daniel E. Mauck, Rebecca B. Hershow, Eleanor Garlow, Jenna Gettings, Dorian Freeman, Anne Marie France, Erica N. Johnson, Agha Ajmal, Dena Elimam, Karrie Reed, Alana Sulka, Jose F. Adame, Jonny F. Andía, Mariana Gutierrez, Mabel Padilla, Nathalie Gonzalez Jimenez, Craig Hayes, Robert P. McClung, Valeria D. Cantos, David P. Holland, Jane Yoon Scott, Alexandra M. Oster, Kathryn G. Curran, Rashida Hassan, Pascale Wortley

**Affiliations:** ^1^Fulton County Board of Health, Atlanta, Georgia; ^2^Division of Infectious Diseases, Department of Medicine, Emory University School of Medicine, Atlanta Georgia; ^3^Epidemic Intelligence Service, CDC; ^4^Division of HIV Prevention, National Center for HIV, Viral Hepatitis, STD, and TB Prevention, CDC; ^5^Georgia Department of Public Health; ^6^Gwinnett, Newton, and Rockdale County Health Department, Lawrenceville, Georgia; ^7^DeKalb County Board of Health, Decatur, Georgia; ^8^Cobb and Douglas Public Health, Marietta, Georgia.

During February 2021–June 2022, the Georgia Department of Public Health (GDPH) detected five clusters of rapid HIV transmission concentrated among Hispanic or Latino (Hispanic) gay, bisexual, and other men who have sex with men (MSM) in metropolitan Atlanta. The clusters were detected through routine analysis of HIV-1 nucleotide sequence data obtained through public health surveillance (*1*,*2*). Beginning in spring 2021, GDPH partnered with health districts with jurisdiction in four metropolitan Atlanta counties (Cobb, DeKalb, Fulton, and Gwinnett) and CDC to investigate factors contributing to HIV spread, epidemiologic characteristics, and transmission patterns. Activities included review of surveillance and partner services interview data,^†^ medical chart reviews, and qualitative interviews with service providers and Hispanic MSM community members. By June 2022, these clusters included 75 persons, including 56% who identified as Hispanic, 96% who reported male sex at birth, 81% who reported male-to-male sexual contact, and 84% of whom resided in the four metropolitan Atlanta counties. Qualitative interviews identified barriers to accessing HIV prevention and care services, including language barriers, immigration- and deportation-related concerns, and cultural norms regarding sexuality-related stigma. GDPH and the health districts expanded coordination, initiated culturally concordant HIV prevention marketing and educational activities, developed partnerships with organizations serving Hispanic communities to enhance outreach and services, and obtained funding for a bilingual patient navigation program with academic partners to provide staff members to help persons overcome barriers and understand the health care system. HIV molecular cluster detection can identify rapid HIV transmission among sexual networks involving ethnic and sexual minority groups, draw attention to the needs of affected populations, and advance health equity through tailored responses that address those needs.

## Investigation and Results

In February 2021, GDPH identified three HIV clusters among Hispanic MSM using molecular analysis of HIV-1 nucleotide sequence data collected through routine surveillance (*1*). In Georgia, clusters are inferred using a genetic distance threshold of 0.005 nucleotide substitutions per site among persons with HIV infection diagnosed during the most recent 3 years, with priority clusters defined as those that include four or more diagnoses during the most recent 12 months. This definition is consistent with evidence of rapid HIV transmission (*1*,*3*). These were the first priority clusters in Georgia comprising ≥40% Hispanic persons. GDPH analysis of HIV surveillance data demonstrated that during 2014–2019, HIV diagnoses among Hispanic adolescents and adults in four metropolitan Atlanta counties increased from 38.9 to 47.1 per 100,000 persons.

After demonstration of persistent growth of the clusters through early 2021, GDPH reviewed partner services interview data and attempted direct outreach to all persons in clusters, including those previously interviewed. However, response was limited, partly attributed to immigration- and deportation-related concerns and limited numbers of bilingual staff members.

In October 2021, CDC began providing remote assistance in analyzing epidemiologic data for investigation activities, and GDPH initiated review of medical charts of persons in clusters. Among 38 persons with available charts, 10 (26%) were primarily Spanish-speaking, and 12 (32%) were from Latin American countries; five (13%) had mental health diagnoses, including depression, anxiety, or bipolar disorder.

In February 2022, GDPH requested CDC assistance in conducting a qualitative assessment with Hispanic MSM community members and service providers to identify barriers to accessing medical and social services and HIV care, as well as simplifying cluster data synthesis and visualization. CDC provided support during March–July 2022. This activity was reviewed by CDC and conducted consistent with applicable federal law and CDC policy.^§^

By June 30, 2022, GDPH detected two additional clusters that included ≥40% Hispanic persons, with additional persons identified among all clusters throughout the investigation period (Figure). The five clusters included 75 persons with HIV, with clusters ranging in size from four to 45 persons. The median age of persons in clusters was 29 years (range = 16–54 years), 56% identified as Hispanic, 96% were assigned male sex at birth, and 81% reported male-to-male sexual contact (Table). Overall, 84% of persons lived in one of the four metropolitan Atlanta counties. Forty percent of diagnoses were from facilities with infectious disease providers who specialize in HIV care, 27% in primary or urgent care settings, 13% in inpatient or emergency department settings, and 11% at health departments. Eighty-five percent of persons in these clusters were virally suppressed^¶^; however, new diagnoses continued to be identified throughout the investigation (Figure).

**FIGURE:**
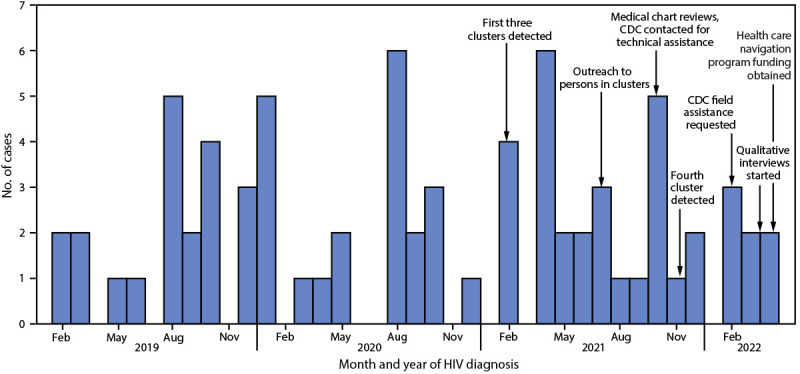
HIV diagnoses by month of diagnosis, and major events during the public health response to five HIV molecular clusters primarily among Hispanic or Latino gay, bisexual, and other men who have sex with men — Metropolitan Atlanta, Georgia, February 2019–April 2022* * Persons in clusters were identified through June 30, 2022, with all identified persons having a diagnosis date of April 30, 2022, or earlier. Persons with more recently diagnosed HIV infection might not yet have been identified as part of a cluster for reasons including time needed to enter HIV care, time for HIV nucleotide sequence to be reported to health department, and molecular analysis schedule.

**TABLE:** Characteristics of persons in five HIV molecular clusters primarily among Hispanic or Latino gay, bisexual, and other men who have sex with men (N = 75) — Metropolitan Atlanta, Georgia, June 2022

Characteristic	No. (%)
**Age, yrs, median (range)**	29 (16–54)
**Received HIV diagnosis during preceding 12 mos**	22 (29)
**Sex at birth**	
Male	72 (96)
Female	3 (4)
**Race and ethnicity**	
Black or African American, non-Hispanic	7 (9)
White, non-Hispanic	15 (20)
Hispanic or Latino	42 (56)
Other, non-Hispanic	11 (15)
**County of residence at diagnosis**	
Cobb	7 (9)
DeKalb	11 (15)
Fulton	10 (13)
Gwinnett	35 (47)
Other	12 (16)
**Born outside the United States**	25 (33)
**HIV transmission category**	
Male-to-male sexual contact	55 (73)
Male-to-male sexual contact and injection drug use	6 (8)
Heterosexual contact	5 (7)
Unknown	9 (12)
**Diagnosis setting**	
Facility with infectious diseases or HIV specialty	30 (40)
Primary or urgent care	20 (27)
Inpatient or emergency facility	10 (13)
Health department	8 (11)
Other*	7 (9)
**Most recent viral load test <200 HIV RNA copies/mL**	64 (85)
**Achieved viral suppression ≤365 days after diagnosis**	69 (92)
**History of HIV PrEP use** ^† ^	4 (5)
**STI diagnosed within 2 mos of HIV diagnosis**	23 (31)
**STI identified during the 12 mos before HIV diagnosis**	4 (5)

**Abbreviations:** PrEP = preexposure prophylaxis; STI = sexually transmitted infection.

* Includes unknown (six) and out of state (one).

^† ^HIV PrEP use ever recorded in partner services interview data.

By June 30, 2022, among 52 persons in clusters eligible for partner services interviews,** 34 (65%) were interviewed, 16 (31%) could not be reached, and two (4%) declined. Among those interviewed, 20 (59%) reported meeting partners online, and four (12%) reported ever having taken HIV preexposure prophylaxis (PrEP).

CDC and health department staff members conducted qualitative interviews with 28 Hispanic MSM and one transgender woman in the four counties and 28 individual or group interviews with 65 medical and social service providers who treated persons in clusters or served Hispanic MSM. Community members were recruited by provider referral, social media, and at bars and clubs. Because multiple attempts had already been made to reach persons in clusters for partner services interviews, further attempts to conduct qualitative interviews were not made for persons in clusters.

Interviewed participants identified barriers to accessing medical and social services, including few Spanish-speaking staff members, limited Spanish language materials, and fear of deportation and other immigration-related concerns. Participants also reported barriers to accessing HIV prevention and care, including stigma toward MSM and persons with HIV because of sexuality-related cultural norms, low levels of awareness about HIV and other sexually transmitted infections because of limited primary care access, limited provision of HIV services in primary and urgent care settings, and limited Hispanic MSM-focused community outreach and marketing.

## Public Health Response

Response activities have included establishing routine coordination meetings between GDPH and metropolitan Atlanta health districts and presenting reports on the investigation to HIV community advisory boards and planning councils. Health districts disseminated Spanish-language HIV prevention materials emphasizing service availability irrespective of immigration status via social media and at venues in zip codes where persons in clusters reside. GDPH established new partnerships with community-based organizations (CBOs) serving Hispanic communities and developed strategies to increase the number of bilingual staff members, including modifying job postings to prioritize hiring bilingual personnel. Health departments and CBOs partnered with academic institutions to engage in implementation science research and obtained federal funding for a culturally concordant outreach and patient navigation program for status-neutral sexual health services. A status-neutral approach provides persons with and without HIV access to comprehensive medical services, including HIV prevention and treatment, and social services depending on their needs.^††^ In addition, in June 2022, the local health districts launched an at-home HIV and sexually transmitted infection self-testing program.^§§^ GDPH is continuing to partner with CDC to implement informatics tools to simplify cluster investigations.

## Discussion

The detection of multiple HIV clusters among Hispanic MSM in metropolitan Atlanta provided evidence of rapid, ongoing HIV transmission and resulted in a multifaceted response involving health departments, CDC, health care providers, and CBOs. The response identified barriers to accessing HIV services among Hispanic MSM in metropolitan Atlanta. Although most persons in clusters had evidence of viral suppression, which prevents sexual HIV transmission, as of June 30, 2022, the clusters were still expanding. This finding indicates potential ongoing transmission among a larger network, which could include persons with undiagnosed HIV infection.

This investigation highlighted the value of molecular HIV cluster detection and response for identifying gaps in services among networks of MSM. Although most large HIV outbreak responses in the United States have focused on persons who inject drugs, male-to-male sexual contact is the primary mode of HIV transmission in most molecular clusters (*4*,*5*). This investigation demonstrated that cluster detection and response can detect rapid HIV transmission and identify population-level gaps in systems involving MSM.

Barriers to accessing HIV services among Hispanic MSM in this investigation included language barriers and immigration- and deportation-related concerns; stigma toward MSM and persons with HIV, often tied to sexuality-related cultural norms; and lack of HIV prevention services in primary and urgent care settings. These findings align with studies identifying access to HIV prevention and care services, language, traditional notions of masculinity, and medical mistrust as barriers to HIV prevention among Hispanic MSM (*6*,*7*). When HIV clusters are detected, it is important to gather data to identify gaps in HIV services so that response efforts can strengthen services for affected populations. Although gaps might already be known, collaborative response efforts can clarify the most important gaps and catalyze new efforts to overcome them such as those described in this response.

The findings in this report are subject to at least three limitations. First, qualitative interviews were conducted among Hispanic MSM community members; thus, findings might not directly reflect the experience of persons in the clusters. Second, because HIV testing and diagnoses substantially declined during the COVID-19 pandemic, cluster size might be underestimated (*8*). Finally, because surveillance and chart review data were incomplete, the proportion of persons in clusters born outside the United States or who were Spanish-speaking might also be underestimated.

This investigation highlights important barriers to and inequalities in HIV prevention services experienced by Hispanic MSM in Georgia because of issues related to language, immigration- and deportation-concerns, and sexuality-related cultural norms. HIV molecular cluster detection has the capability to identify rapid HIV transmission in a new demographic group and advance health equity through expanded and tailored resources for HIV prevention and care.

**Summary**

**What is already known about this topic?**

Molecular HIV clusters provide evidence of rapid transmission.

**What is added by this report?**

In 2021, molecular HIV analysis in Georgia identified clusters of rapid HIV transmission among Hispanic or Latino (Hispanic) gay, bisexual, and other men who have sex with men (MSM) in metropolitan Atlanta. A multicomponent investigation identified factors that might limit access to HIV services, including language barriers, immigration- and deportation-related concerns, and sexuality-related cultural norms. Health departments, providers, and community-based organizations collaborated to address these barriers.

**What are the implications for public health practice?**

Hispanic MSM can face important barriers to accessing HIV services. Detecting and responding to HIV clusters among MSM can mobilize resources to strengthen services and improve health equity.

## Footnotes

* These authors contributed equally to this report.

† Partner services interviews are completed by health departments for eligible persons with a newly diagnosed HIV infection and, potentially, after a person with known HIV infection is identified as part of a cluster. Interviews are completed to ensure that persons with HIV infection are linked to care and to obtain information about their sexual partners, who can receive notification of their potential exposure and services, including testing and HIV preexposure prophylaxis.

§ 45 C.F.R. part 46, 21 C.F.R. part 56; 42 U.S.C. Sect. 241(d); 5 U.S.C. Sect. 552a; 44 U.S.C. Sect. 3501 et seq.

¶ HIV suppression is defined as HIV viral load <200 copies of HIV RNA per mL of blood in the preceding year in Georgia Department of Public Health HIV surveillance data.

** Persons who received an HIV diagnosis from a health department or entity funded by a health department are eligible for partner services interviews in Georgia.

††
https://www.cdc.gov/hiv/policies/data/status-neutral-issue-brief.html

§§ https://stophivatl.org/free-hiv-sti-home-test/

## References

[1] Oster AM, France AM, Panneer N, Identifying clusters of recent and rapid HIV transmission through analysis of molecular surveillance data. J Acquir Immune Defic Syndr 2018;79:543–50. 10.1097/QAI.0000000000001856 30222659 PMC6231979

[2] Oster AM, Lyss SB, McClung RP, HIV cluster and outbreak detection and response: the science and experience. Am J Prev Med 2021;61(Suppl 1):S130–42. 10.1016/j.amepre.2021.05.029 34686282 PMC12599833

[3] Kosakovsky Pond SL, Weaver S, Leigh Brown AJ, Wertheim JO. HIV-TRACE (TRAnsmission Cluster Engine): a tool for large scale molecular epidemiology of HIV-1 and other rapidly evolving pathogens. Mol Biol Evol 2018;35:1812–9. 10.1093/molbev/msy016 29401317 PMC5995201

[4] Lyss SB, Buchacz K, McClung RP, Asher A, Oster AM. Responding to outbreaks of human immunodeficiency virus among persons who inject drugs—United States, 2016–2019: perspectives on recent experience and lessons learned. J Infect Dis 2020;222(Suppl 5):S239–49. 10.1093/infdis/jiaa112 32877545

[5] Perez SM, Panneer N, France AM, Clusters of rapid HIV transmission among gay, bisexual, and other men who have sex with men—United States, 2018–2021. MMWR Morb Mortal Wkly Rep 2022;71:1201–6. 10.15585/mmwr.mm7138a1 36136909 PMC9531569

[6] Horridge DN, Oh TS, Alonzo J, Barriers to HIV testing within a sample of Spanish-speaking Latinx gay, bisexual, and other men who have sex with men: implications for HIV prevention and care. Health Behav Res 2019;2. 10.4148/2572-1836.1069 31799502 PMC6889883

[7] Kimball D, Rivera D, Gonzales M 4th, Blashill AJ. Medical mistrust and the PrEP cascade among Latino sexual minority men. AIDS Behav 2020;24:3456–61. 10.1007/s10461-020-02916-z 32405726 PMC7665998

[8] DiNenno EA, Delaney KP, Pitasi MA, HIV testing before and during the COVID-19 pandemic—United States, 2019–2020. MMWR Morb Mortal Wkly Rep 2022;71:820–4. 10.15585/mmwr.mm7125a2 35737573

